# A study of the use of carbamazepine, pregabalin and alpha lipoic acid in patients of diabetic neuropathy

**DOI:** 10.1186/2251-6581-13-62

**Published:** 2014-05-27

**Authors:** Niral Patel, Vishal Mishra, Prakruti Patel, Ram K Dikshit

**Affiliations:** 1 Medwise Pharmaceuticals, Ahmedabad, India; 2 GMERS medical colloge, Patan, India; 3 B J Medical College, Civil hospital, Ahmedabad, Gujarat, India; 4 Department of Pharmacology, GCS Medical College, Ahmedabad, Gujarat, India

**Keywords:** Diabetic neuropathy, Pregabalin, Carbamazepine, Alpha lipoic acid

## Abstract

**Background:**

Diabetic peripheral neuropathy (DPN) is a common, symptomatic, long-term complication of diabetes mellitus. Many of the agents used to treat DN have not been compared with each other. This study was, therefore, undertaken to compare the efficacy and safety of carbamazepine, pregabalin and alpha-lipoic acid in diabetic neuropathy patients.

**Methods:**

This was a prospective, observational study. The patients were categorized into three groups, Group I included those patients who were prescribed carbamazepine while group II included those on pregabalin and group III patients received alpha-lipoic acid. Each patient was followed up at every month for total duration of 6 months. Demographic details, presenting symptoms, history of diabetes, laboratory values pertaining to diabetes (Fasting blood sugar, Post prandial blood sugar and HbA1c) were recorded. Intensity of pain, using a visual analogue scale (VAS), diabetic neuropathy symptom (DNS) score and diabetic neuropathy examination (DNE) score were assessed at baseline and then at each monthly follow-up. Nerve conduction velocity (NCV) was also measured at baseline and then at the end of 3 and 6 months.

**Results:**

A total of 101 patients were enrolled out of them 96 completed the study. Regarding VAS, the number of patients having pain was reduced substantially however, the speed and the quantum of this reduction were best in group II (pregabalin). Regarding DNS, also group II showed the best response in terms of number of patients as well as the speed of improvement. The results also imply that the relief from diabetic neuropathy (as per DNE score) is superior with pregabalin administration. However, no improvement in NCV was evident in any group.

**Conclusion:**

Results of this study suggest that treatment with pregabalin gives faster and better improvement in diabetic neuropathy.

## Background

Diabetic peripheral neuropathy (DPN) is a common, symptomatic, long-term complication of type 1 and 2 diabetes mellitus. At the initial diagnosis, 10% of the patients already experience pain, and about 50% are afflicted with this complication after 25 years of the disease [1]. Neuropathies are characterized by a progressive loss of nerve fibers, which may affect both the somatic as well as autonomic nervous system. The clinical features vary immensely, and patients may present a variety of complaints ranging from paraesthesias to disabling neuropathy and cardiac arrhythmias. Mechanisms involved behind nerve damage are persistent hyperglycemia, microvascular insufficiency, oxidative and nitrosative stress, defective neurotrophism, and autoimmune nerve destruction. DN is the leading cause of diabetes-related hospital admissions and non-traumatic amputations [2,3]. It leads to a major physical disability, poor quality of life [4], high mortality [3], and an estimated total annual cost of $22 billion [5].

A large number of randomized clinical trials have been conducted to assess the efficacy of various therapeutic agents but the results have been disappointing, most probably due to the complexity of mechanisms involved in its pathogenesis [3,6]. Therefore, till date no effective treatment exists for DN, other than the control of hyperglycemia [7,8]. Several drugs have been used with varying degree of success including antidepressants (primarily tricyclic antidepressants or TCAs) and anti-epileptics (carbamazepine, gabapentin, pregabalin, lamotrigine, sodium valporate and topiramate). Other drugs like α-lipoic acid, baclofen, levodopa, methcobalamin, bupropion, are also claimed to be beneficial. Many of the agents used to treat DN have not been compared with each other. Also, the end points in many of the studies have varied, making it difficult to compare the treatments [9]. This study therefore aimed to compare the efficacy and safety of carbamazepine (older treatment), pregabalin (currently preferred) and alpha lipoic acid (newer drug) in the treatment of diabetic neuropathy.

## Materials and methods

This was a prospective, observational study conducted at the Diabetic outpatient department, of a tertiary care, teaching hospital. The study was carried out over a period of 21 months from October 2010 to June 2012. Approval from The Institutional Ethics Committee (Institutional Ethics Committee, BJMC, Ahmedabad) was obtained. The enrolled patients were followed up monthly for 6 months. They were categorized into three groups according to treatment prescribed by the physician. Group I included those patients of diabetic neuropathy (DN) who were prescribed carbamazepine while group II included those on pregabalin and group III patients received alpha-lipoic acid. Demographic details, presenting symptoms, history of diabetes, laboratory values pertaining to diabetes (FBS, PPBS and HbA1c) were recorded. Several tests were conducted to assess the severity of DN and its amelioration in response to the treatment given. Intensity of pain, using a visual analogue scale (VAS), diabetic neuropathy symptom score (DNS) and diabetic neuropathy examination score (DNE) were assessed at baseline and then at each monthly follow-up. Nerve conduction velocity (NCV) was measured at baseline and then at the end of 3 and 6 months.

The VAS used for measuring the pain intensity was a 10-cm horizontal line that ranged from no pain (0) to worst pain (10) imaginable with mild, moderate, severe and very severe grades [10]. DNS consists of four questions related to symptoms of diabetic neuropathy. At every visit, patients were asked if they had experienced any of these symptoms during the last two weeks. For each question a positive reply was counted as one point and negative as zero. If total score (out of 4) was one or more, diabetic neuropathy was considered to be present [11]. DNE score (diabetic neuropathy examination score) consists of eight parameters including two for muscle strength, one for tendon reflex and five for sensations. A total score (out of 16) was recorded for each patient and those having it > 3 were diagnosed to be suffering from diabetic neuropathy [12]. NCV (nerve conduction velocity) was evaluated on both tibial and peroneal nerves on both the legs.

### Data analysis

The value of each test was compared with baseline and between groups using Fisher’s exact test andone way analysis of variance (ANOVA). Adverse drug reactions were recorded and analysed.

## Results

A total of 101 patients were enrolled for the study. Out of them 96 completed the study (44 male and 52 female) whereas 5 patients were lost to follow up due to non compliance. The mean age of patients in group I (carbamazepine) was 54.5 ± 8.3 years, 54.8 ± 7.2 years in group II (pregabalin) and 57.3 ± 8 years in group III (alpha-lipoic acid). The average duration of diabetes was 10.43 ± 3.42 years (no statistical difference between groups). There were 51 patients taking oral hypoglycemic drugs, 12 patients received insulin while 33 patients were receiving both. Hypertension was the most common concomitant disease (n = 47). At baseline, the most common symptom of diabetes was polyuria (78.8, 84.3 and 70.9% patients in group I, II and III respectively) followed by polydypsia (45.4, 34.3 and 45.2% patients in group I, II and III, respectively). A significant (*p* < 0.05) reduction in number of patients having polyuria (57.7, 77.7 and 59.1%in Group I, II and II respectively) and polydypsia (93.3, 72.7 and 78.6% in Group I, II and III respectively) was observed in each group at the end of the study. At baseline the most common presenting symptom of diabetic neuropathy (DN) was burning/aching pain in one or both lower limbs reported by all patients. Pricking sensation in one or both lower limbs was the next common symptom reported by majority of the patients in all three groups (72.8% in group I, 81.3 in group II and 71%in group III). Improvement in symptoms of DN was noticeable in all three groups but it was relatively faster and better in patients of group II (pregabalin) starting from first follow up itself. In all the groups, a significant (*p* < 0.05) improvement in FBS, PPBS and HbA1c was also seen in with effect from first or second follow up and was maintained until the end of study.

### Pain

Severity of pain (as assessed by VAS) was classified into six categories, 0 = no pain, 1–2 = mild, 3–4 = moderate, 5–6 = severe, 7–8 = very severe and 9–10 = worst pain. A detailed classification of patients in each category is given in Table 1. At baseline, no patient was classified as ‘no pain’ or ‘worst pain’ in all three groups. A significant (*p* < 0.05) number of patients had ‘no pain’ with effect from 3rd follow up in group I, 1st follow up in group II and 2nd follow up in group III. A further analysis of patients having ‘no pain’ revealed that group II had significantly (*p* < 0.05) higher rate of improvement as compared to other groups up to fourth follow up, while there was no significant difference between groups I and III. It is apparent that while the number of patients having mild, moderate, severe and very severe pain was reduced substantially by the end of the study (as compared to baseline), the speed and the quantum of this reduction was best in group II (pregabalin).

**Table 1:** Distribution of patients suffering from neuropathic pain

	**Severity of pain (as per VAS score)**^**#**^	**Number of patients**						
		**Baseline**	**1st FU**	**2nd FU**	**3rd FU**	**4th FU**	**5th FU**	**6th FU**
**Group I (n=33) (Carbamazepine)**	**No pain (0)**	0	3	5	12**	22**	27**	28**
	**Mild pain (1–2)**	6	8	12	10	8	4	4
	**Moderate pain (3–4)**	14	14	12	8	2**	2**	1**
	**Severe pain (5–6)**	8	4	3	2	1*	0**	0**
	**Very severe pain (7–8)**	5	4	1	1	0	0	0
**Group II (n=32) (Pregabalin)**	**No pain (0)**	0	8**	13**	18**	27**	30**	32**
	**Mild pain (1–2)**	6	8	11	12	4	2	0
	**Moderate pain (3–4)**	22	15	8**	2**	1**	0**	0**
	**Severe pain (5–6)**	3	1	0	0	0	0	0
	**Very severe pain (7–8)**	1	0	0	0	0	0	0
**Group III (n=31) (Alpha lipoic acid)**	**No pain (0)**	0	5	8*	14**	23**	25**	29**
	**Mild pain (1–2)**	8	14	14	11	6	5	2
	**Moderate pain (3–4)**	16	10	9	6*	2**	1**	0**
	**Severe pain (5–6)**	5	1	0	0	0	0	0
	**Very severe pain (7–8)**	2	1	0	0	0	0	0

FU= Follow-Up, *p<0.05, **p < 0.01 as compared to baseline (Fisher’s exact test), ^#^no patient was classified as ‘worst pain (9–10)’ in any group at any time, VAS= visual analogue scale.

### Symptomatic improvement

**Figure 1:**
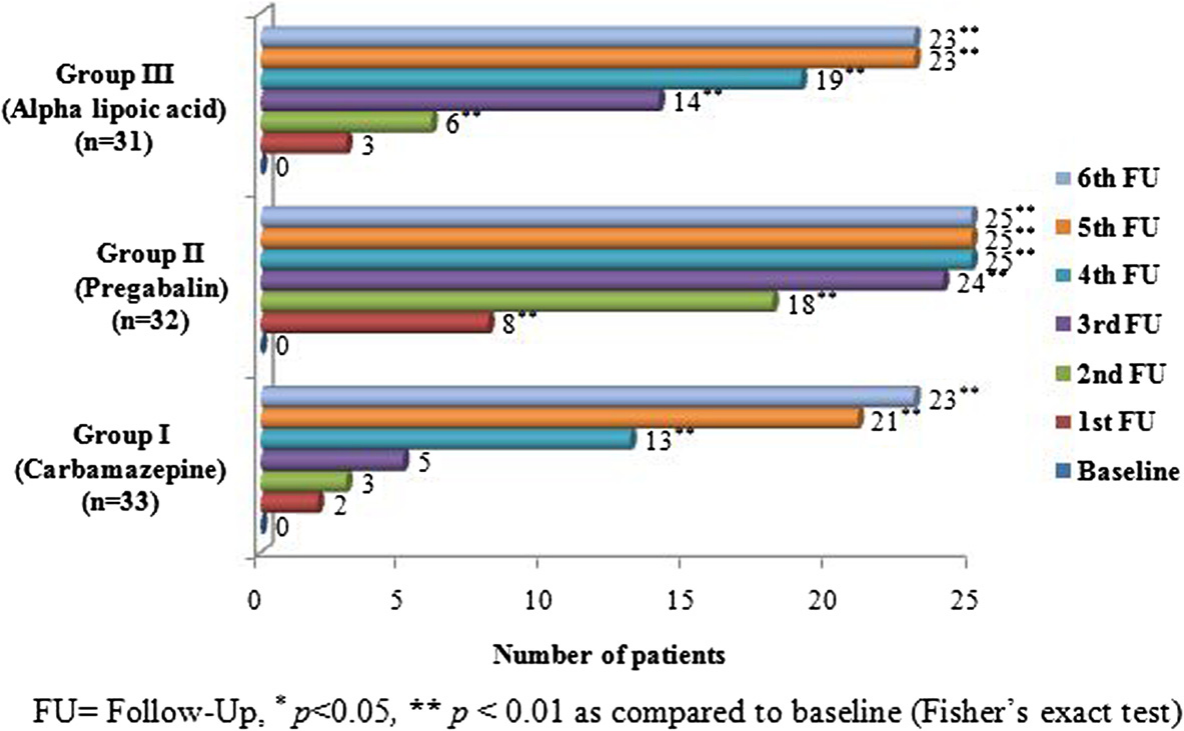
Number of symptom free patients as per diabetic neuropathy symptom (DNS) score.

The number of patients in each group with the zero DNS score (depicting total freedom from the symptoms of diabetic neuropathy) was also calculated at every follow up. An approximately similar number of patients were observed to be having zero score by the end of this study. However, group II showed the best response in terms of number of patients as well as the speed of improvement as compared to group I and III. The difference in improvement between group II and group I and III was statistically significant (*p* < 0.05) (Figure 1).

### Relief from diabetic neuropathy

**Figure 2:**
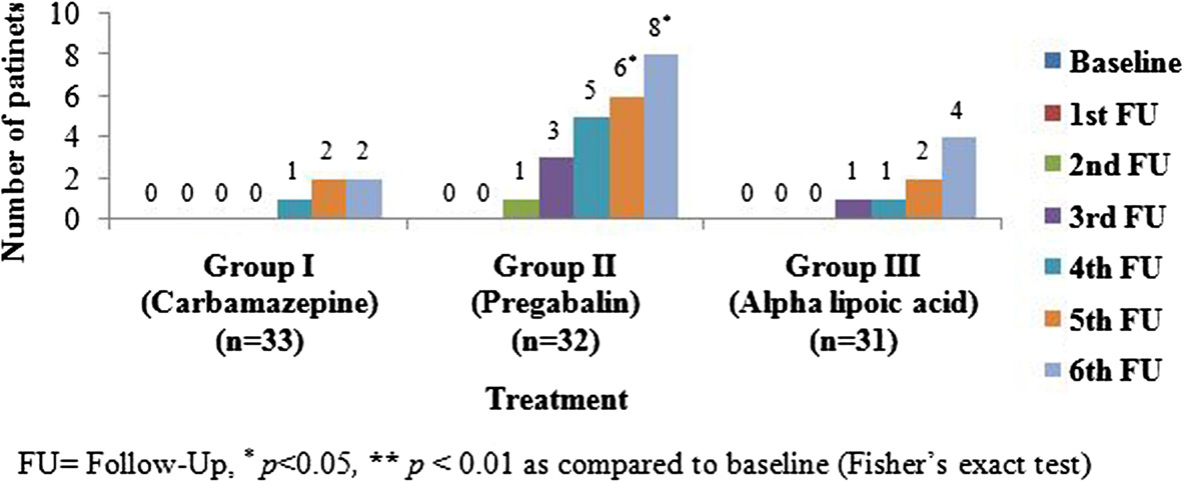
Number of patients with less than free score on diabetic neuropathy examination (‘no neuropathy’).

DNE score less than three is considered as absence of diabetic neuropathy. At every follow up number of patients having DNE score less than three was noted. A significant (*p* < 0.05) improvement was evident only in group II at fifth and sixth follow ups (6 and 8 patients out of 32 respectively). It was observed that the number of patients with absence of DN (score less than 3) was significantly more in group II than group I and III. The difference noted between group I and III was statistically insignificant (Figure 2). The results imply that the relief from diabetic neuropathy (as per DNE score) is superior with pregabalin administration as compared to carbamazepine and alpha lipoic acid.

#### Nerve conduction velocity

No improvement in NCV was evident in any group at 3rd or 6th follow ups. These results showed that there was no change in basic pathophysiology of diabetic neuropathy in any of the treatment group.

### Correlation between glycemic control and diabetic neuropathy

**Figure 3:**
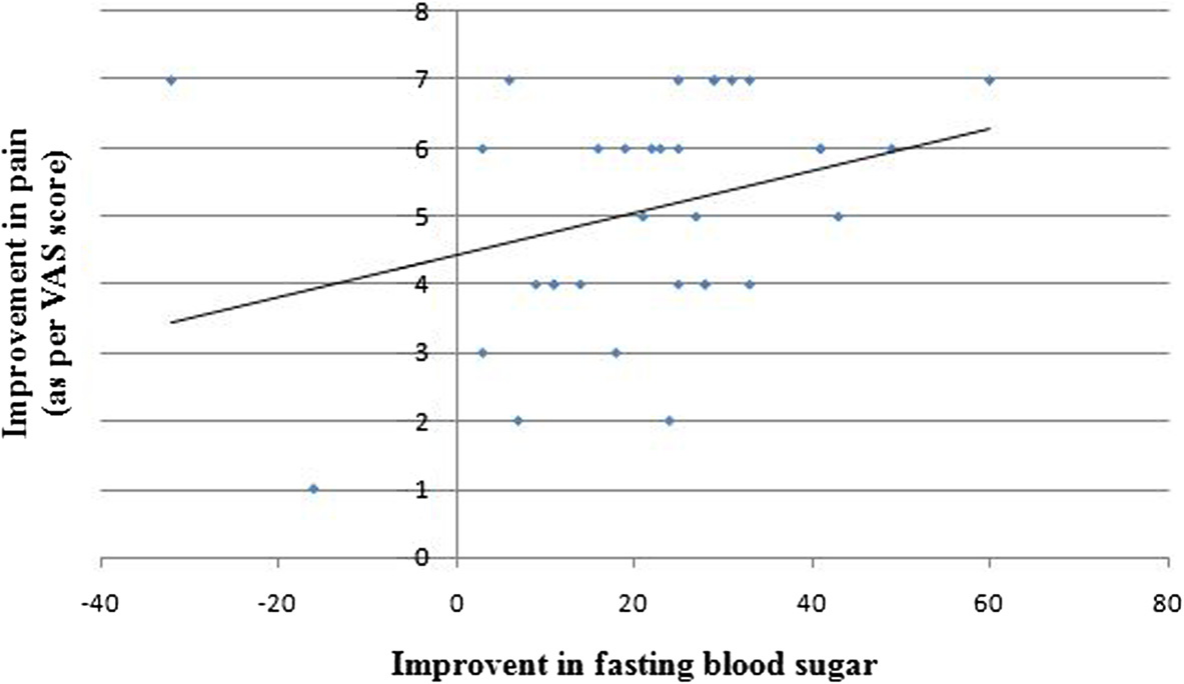
Correlation betweeen fasting blood sugar and degree of pain in patients suffering from neurophy treated with carbamazepine.

A correlation between glycemic control (as assessed by FBS) and parameters of DN (VAS, DNS and DNE) was studied. Only group I showed a significant (p < 0.05) correlation between FBS and VAS (correlation coefficient (r) was 0.35, confidence interval (95%) 0.01 to 0.6 and coefficient of determination (r [2]) 0.12). This means that the improvement in FBS correlates with improvement in VAS in patients treated with carbamazepine (Figure 3).

Regarding safety, the drug therapy was well tolerated in all groups. Only three (3) adverse drug reactions were reported during the study period. Two patients developed rashes with carbamazepine while one had somnolence with pregabalin. These ADRs were non-serious in nature and the patients recovered completely.

## Discussion

Diabetic neuropathy is a common long term complication of diabetes mellitus affecting nearly 50% of the patients [13]. The mean age of the patients in our study (n = 96) was 55.28 ± 7.85 years which is similar to other studies [14-16]. It is reported that the advancing age increases the incidence of neuropathy. The average duration of diabetes in our study was 10 years which is not very long yet correlates well with previous reports [16,17]. Prolonged exposure of peripheral nerves to hyperglycemia in long standing diabetics predisposes them to development of neuropathy. The role of glycemic control in development as well as progression of DN is supported by the available evidence [18]. In our study, the most common presenting symptom of DN was burning/aching pain in one or both lower limbs. Pricking sensation was the next common symptom followed by unsteadiness on walking and numbness in that order. A significant (*p* < 0.05) improvement in burning/aching pain and pricking sensation was found in all the treatment groups at various follow ups. However, patients treated with pregabalin showed the best response in terms of rate (100%) and speed of improvement. While a similar results for early and sustained improvement with pregabalin have been reported [19], a meta-analysis of seven randomized trials has also described the similar findings [20].

We conducted several specialized tests to evaluate the status of pain (visual analogue scale), other symptoms (diabetic neuropathy symptom score) and signs (diabetic neuropathy examination) of diabetic neuropathy as well as the peripheral nerve physiology (nerve conduction velocity). As known, pain and sensation abnormality are the classical symptoms of DN. We evaluated them by using visual analogue scale (VAS) and neuropathy symptoms score (DNS) respectively. VAS is a reliable, valid and widely used test to assess the pain intensity. According to VAS score six categories can be classified ranging from no pain (0) to worst possible pain (9–10) [21]. Our results using VAS confirmed the clinical observations about pain in DN described above. Complete relief from pain (‘no pain’) should be considered to be more important than a simple reduction in pain intensity over long term. We found that the number of patients in ‘no pain’ category at every follow up increased significantly (*p* < 0.05) in all the groups. However, the reduction in mean VAS scores and in number of patients having ‘no pain’ was relatively better with pregabalin. It is difficult to explain a superior response to pregabalin. It, however, appears that a unique mechanism action of pregabalin may be responsible for this. Pregabalin blocks alpha 2 delta protein, an auxillary subunit of voltage gated calcium channel. It also reduces the synaptic release of several neurotransmitters apparently by the same mechanism which possibly results in the reduction of neuronal excitability and ultimately the pain [22]. On the other hand, the diabetic neuropathy symptom score is also a validated and widely accepted scoring system for screening of diabetic neuropathy [23]. It comprises four questions in relation to the symptoms of diabetic neuropathy. It is calculated on the basis of total positive replies. DNS greater than one is considered as presence of diabetic peripheral neuropathy. This score is also valid to be used for predictive purpose or prognostics [11]. Number of patients achieving zero score (no symptoms) were similar with pegabalin and alpha lipoic acid (78 vs 74%). Our results imply that although pregabalin shows a faster rate of improvement, it is otherwise therapeutically equivalent to alpha lipoic acid. It also appears that alpha lipoic acid may have a lesser effect on pain as compared to pregabalin but its anti-inflammatory and antioxidant actions may contribute significantly to an all-round improvement in the symptoms of DN. To sum up the results of VAS and DNS, it can be stated that the reduction in pain (as per VAS) is superior with pregabalin (as compared to alpha-lipoic acid and carbamazepine). However, the reduction in symptom score (as per DNS) is same with pregabalin and alpha lipoic acid. Carbamazepine shows a poor rate and extent of improvement.

Another special test conducted during this study was diabetic neuropathy examination (DNE) score. This is a sensitive and well-recognized scoring system that is fast and easy to perform in clinical practice. Its predictive value is high both for screening and prognosis of diabetic neuropathy [11]. It consists of examination of various sensations, reflexes and muscle strength in lower limbs. Total score greater than 3 (out of 16) is considered as presence of diabetic peripheral neuropathy. The mean DNE score in all three groups in our study was nearly 8 at baseline. The number of patients with <3 score (no DN) showed statistically significant increase only in the pregabalin group. This indicates the superiority of pregabalin over other drugs. The reason for better improvement with pregabalin may be due to its multiple actions as antiepileptic and anxiolytic agent in addition to its analgesic action. An additional central nervous system effect is also likely which may be beneficial in improving the parameters of DNE. Carbamazepine also has central actions but it showed a lesser improvement compared to pregabalin. It may be noted here that carbamazepine acts through sodium channels while pregabalin acts predominantly on calcium channels. The action on calcium channel modulates the release of various neurotransmitters in CNS [24] and this may result in a better effect of pregabalin. However, despite the lack of a central action alpha lipoic acid also showed a beneficial effect which was better than carbamazepine which may be due to its additional anti-inflammatory and antioxidant action [25,26]. It is summed up that the improvement in sensations, reflexes and muscle strength in patients of DN was observed to be best with pregabalin (as per DNE score). The unique effect of pregabalin on calcium channel and thus on neurotransmitter release may be responsible for this.

We also conducted the tests on nerve conduction velocity in patients of DN in this study. Nerve conduction velocity (NCV) is the most sensitive and specific method for detection of DN. The use of NCV is recommended for early diagnosis as well as follow-up in DN [27]. Comparing the improvement in NCV at the end of study showed no significant change in all three groups. Perhaps a short duration of follow up for only 6 months may be the reason for this. However, failure of improvement in NCV by pregabalin has been reported by others also [28]. A study conducted in India has also described the similar findings for carbamazepine [29]. On the other hand, a meta-analysis conducted for alpha-lipoic acid has showed significant improvement in NCV [30]. In this analysis a higher dose of alpha-lipoic acid (300–600 mg, iv) was reported to show the benefit while a lower dose 200 mg orally has only been used in our study.

A strong correlation between improvement in glycemic control and diabetic neuropathy has been reported in various trials [18]. In present study, a significant (*p* < 0.05) improvement in various tests of diabetic neuropathy (VAS, DNS and DNE) and glycemic control (FBS) was observed separately with all three treatments. It gives an impression that the improvement in DN may have been due to the improved glycemic control with anti-diabetic drug therapy. On further analysis, however, it was observed that a significant correlation between FBS and the parameters of DN did not exist in any of the groups. A small exception was the correlation between FBS and improved VAS score in patient treated with carbamazepine. It is therefore opined that the improvement in DN in any of the groups was not due to a better glycemic control but it rather appears to be due to the specific treatment (carbamazepine, pregabalin and alpha lipoic acid) received by the patients. It is also concluded that the medication received by the patients in our study was very well tolerated as very few adverse drug reactions of mild variety were reported during this period.

## Conclusion

Results of this study suggest that treatment with pregabalin is associated with faster and better improvement in diabetic neuropathy. Effectiveness of alpha lipoic acid appears to be equivalent to pregabalin. However, carbamazepine appeared to be inferior to the other two drugs. Use of pregabalin as first line therapy in diabetic neuropathy is, therefore, justified and recommended.

## Competing interests

There is no competing interest of the author.

## Authors’ contributions

NM carried out the study, PP, VM and RKD carried out the initial designing of study, reviewing and final drafting activities. All authors read and approved the final manuscript.

## References

[B1] Fedele D Comi G Coscelli C Cucinotta D Feldman EL Ghirlanda G Greene DA Negrin P Santeusanio F A multicenter study on the prevalence of diabetic neuropathy in Italy. Italian Diabetic Neuropathy Committee Diabetes Care 1997 20 5 836 843 10.2337/diacare.20.5.836 9135952

[B2] Pirart J Diabetes mellitus and its degenerative complications: a prospective study of 4,400 patients observed between 1947 and 1973 (3rd and last part) Diabetes Metab 1977 3 245 256 598565

[B3] Boulton AJ Vinik AI Arezzo JC Bril V Feldman EL Freeman R Malik RA Maser RE Sosenko JM Ziegler D American Diabetes Association Diabetic neuropathies: a statement by the American Diabetes Association Diabetes Care 2005 28 9 56 62 10.2337/diacare.28.4.956 15793206

[B4] Vileikyte L Leventhal H Gonzalez JS Peyrot M Rubin RR Ulbrecht JS Garrow A Waterman C Cavanagh PR Boulton AJ Diabetic peripheral neuropathy and depressive symptoms: the association revisited Diabetes Care 2005 28 2378 2383 10.2337/diacare.28.10.2378 16186266

[B5] American diabetes association. Living with diabetes. High blood pressure (hypertension) Available from: http://www.diabetes.org/living-with-diabetes/complications/high-blood-pressure-hypertension.html

[B6] Pop-Busui R Sima A Stevens M Diabetic neuropathy and oxidative stress Diabetes Metab Res Rev 2006 22 257 273 10.1002/dmrr.625 16506271

[B7] Ohkubo Y Kishikawa H Araki E Miyata T Isami S Motoyoshi S Kojima Y Furuyoshi N Shichiri M Intensive insulin therapy prevents the progression of diabetic microvascular complications in Japanese patients with non-insulin-dependent diabetes mellitus: a randomized prospective 6-year study Diabetes Res Clin Pract 1995 28 103 117 10.1016/0168-8227(95)01064-K 7587918

[B8] Diabetes Control and Complications Trial Research Group The effect of intensive treatment of diabetes on the development and progression of long-term complications in insulin-dependent diabetes mellitus N Engl J Med 1993 329 977 986 8366922 10.1056/NEJM199309303291401

[B9] Huizinga M Peltier A Painful diabetic neuropathy: a management-centered review Clin Diabetes 2007 25 6 15 10.2337/diaclin.25.1.6

[B10] Dauphin A Guillemin F Virion J Briançon S Bias and precision in visual analogue scales: a randomized controlled trial Am J Epidemiol 1999 150 10 1117 1127 10.1093/oxfordjournals.aje.a009937 10568628

[B11] Meijer JW Smit AJ Sonderen EV Groothoff JW Eisma WH Links TP Symptom scoring systems to diagnose distal polyneuropathy in diabetes: the Diabetic Neuropathy Symptom score Diabet Med 2000 19 11 962 965 12421436 10.1046/j.1464-5491.2002.00819.x

[B12] Meijer JW Sonderen E Blaauwwiekel EE Smit AJ Groothoff JW Eisma WH Links TP Diabetic neuropathy examination: a hierarchical scoring system to diagnose distal polyneuropathy in diabetes Diabetes Care 2000 23 750 753 10.2337/diacare.23.6.750 10840990

[B13] Tesfaye S Boulton AJ Dyck PJ Freeman R Horowitz M Kempler P Lauria G Malik RA Spallone V Vinik A Bernardi L Valensi P Toronto Diabetic Neuropathy Expert Group Diabetic neuropathies: update on definitions, diagnostic criteria, estimation of severity, and treatments Diabetes Care 2010 33 2285 2293 10.2337/dc10-1303 20876709 PMC2945176

[B14] Dyck PJ Davies JL Wilson DM Service FJ Melton LJ III O’Brien P Risk factors for severity of diabetic polyneuropathy: intensive longitudinal assessment of the Rochester Diabetic Neuropathy Study cohort Diabetes Care 1999 22 9 1479 1486 10.2337/diacare.22.9.1479 10480512

[B15] Dutta A Naorem S Singh T Wangjam K Prevalence of peripheral neuropathy in newly diagnosed type 2 diabetes Int J Diabetes Dev Ctries 2005 25 30 33 10.4103/0973-3930.26756

[B16] Pradeepa R Rema M Vignesh J Deepa M Deepa R Mohan V Prevalence and risk factors for diabetic neuropathy in an urban south Indian population: the Chennai Urban Rural Epidemiology Study (CURES‒55) Diabet Med 2008 25 407 412 10.1111/j.1464-5491.2008.02397.x 18294224

[B17] Janghorbani M Rezvanian H Kachooei A Ghorbani A Chitsaz A Izadi F Amini M Peripheral neuropathy in type 2 diabetes mellitus in Isfahan, Iran: prevalence and risk factors Int J Diabetes Metab 2006 14 126 133 10.1111/j.1600-0404.2006.00716.x 17083338

[B18] American Diabetes Association Diabetes Care 2002 25 1 28 32

[B19] Lesser H Sharma U LaMoreaux L Poole RM Pregabalin relieves symptoms of painful diabetic neuropathy Neurology 2004 63 11 2104 2110 10.1212/01.WNL.0000145767.36287.A1 15596757

[B20] Freeman R Durso-Decruz E Emir B Efficacy, safety and tolerability of pregabalin treatment for painful diabetic peripheral neuropathy: findings from seven randomized, controlled trials across a range of doses Diabetes Care 2008 31 1448 1454 10.2337/dc07-2105 18356405 PMC2453685

[B21] Bodian CA Freedman G Hossain S Eisenkraft JB Beilin Y The visual analog scale for pain: clinical significance in postoperative patients Anesthesiology 2001 95 1356 1361 10.1097/00000542-200112000-00013 11748392

[B22] Taylor CP Angelotti T Fauman E Pharmacology and mechanism of action of pregabalin: the calcium channel alpha2-delta (alpha2-delta) subunit as a target for antiepileptic drug discovery Epilepsy Res 2007 73 137 150 10.1016/j.eplepsyres.2006.09.008 17126531

[B23] Dyck PJ Detection, characterization and staging of polyneuropathy: assessed in diabetics Muscle Nerve 1988 11 21 32 10.1002/mus.880110106 3277049

[B24] Hamandi K Sander JW Pregabalin: a new antiepileptic drug for refractory epilepsy Seizure 2006 15 2 73 78 10.1016/j.seizure.2005.11.005 16413993

[B25] Brasier AR The NF-kappaB regulatory network Cardiovasc Toxicol 2006 6 111 130 10.1385/CT:6:2:111 17303919

[B26] Shay P Moreau RF Smith EJ Hagen TM Is alpha-lipoic acid a scavenger of reactive oxygen species in vivo? Evidence for its initiation of stress signaling pathways that promote endogenous antioxidant capacity IUBMB Life 2008 60 362 367 10.1002/iub.40 18409172

[B27] Kong X Lesser EA Potts FA Gozani SN Utilization of nerve conduction studies for the diagnosis of polyneuropathy in patients with diabetes: a retrospective analysis of a large patient series J Diabetes Sci Technol 2008 2 2 268 274 10.1177/193229680800200217 19885354 PMC2771502

[B28] Arezzo JC Rosenstock J LaMoreaux L Pauer L Efficacy and safety of pregabalin 600 mg/d for treating painful diabetic peripheral neuropathy: a double-blind placebo-controlled trial BMC Neurol 2008 8 33 10.1186/1471-2377-8-33 18796160 PMC2565674

[B29] Chakrabarti AK Samantaray SK Diabetic peripheral neuropathy: nerve conduction studies before, during and after carbamazepine therapy Aust NZJ Med 1976 6 565 568 10.1111/j.1445-5994.1976.tb03996.x 1071542

[B30] Han T Bai J Liu W Hu Y A systematic review and meta-analysis of alpha lipoic acid in the treatment of diabetic peripheral neuropathy Eur J Endocrinol 2012 167 465 471 10.1530/EJE-12-0555 22837391

